# Supplementary material for: Silver Nanoparticles Biocomposite Films with Antimicrobial Activity: In Vitro and In Vivo Tests
Source: Int J Mol Sci. 2022 Sep 14;23(18):10671. doi: 10.3390/ijms231810671 (PMC9503464; doi:10.3390/ijms231810671)
Supplement: Supplementary file 1 [file ijms-23-10671-s001.zip › ijms-1854163-SI.pdf]

Article

Silver Nanoparticles Biocomposite Films with Antimicrobial Activity: In Vitro and In Vivo Tests

Anca Niculina Cadinoiu [1], Delia Mihaela Rata [1,*], Oana Maria Daraba [1], Daniela Luminita Ichim [1], Irina Popescu [2], Carmen Solcan [3] and Gheorghe Solcan [3]

[1] Faculty of Medical Dentistry, "Apollonia" University of Iasi, 700511 Iasi, Romania
[2] Petru Poni Institute of Macromolecular Chemistry, 700487 Iasi, Romania
[3] Faculty of Veterinary Medicine, "Ion Ionescu de la Brad" Iasi University of Life Sciences, 700489 Iasi, Romania
* Correspondence: delia.rata@univapollonia.ro

Supplementary Materials:

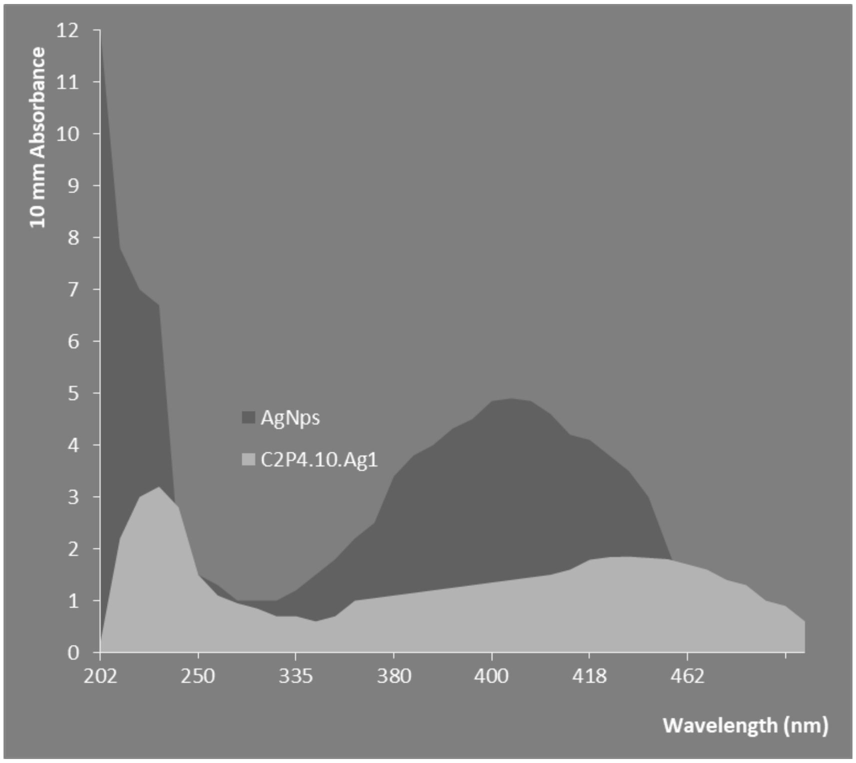

Figure S1. UV-Vis spectroscopy results for AgNps and C2P4.10.Ag1 film samples after 24 h of stirring in water
